# P-2141. Candidemia after Solid Organ Transplantation

**DOI:** 10.1093/ofid/ofaf695.2304

**Published:** 2026-01-11

**Authors:** Safah Khan, Lana Hasan, Jessica Lum, Zachary Yetmar

**Affiliations:** Cleveland Clinic Foundation, Cleveland, Ohio; Cleveland Clinic, Cleveland, Ohio; Cleveland Clinic Foundation, Cleveland, Ohio; Cleveland Clinic, Cleveland, Ohio

## Abstract

**Background:**

Invasive fungal infections, most commonly due to *Candida*, are a dreaded complication after solid organ transplantation (SOT). This study aims to characterize the epidemiology of candidemia in SOT recipients at a tertiary transplant center.

**Table 1:**
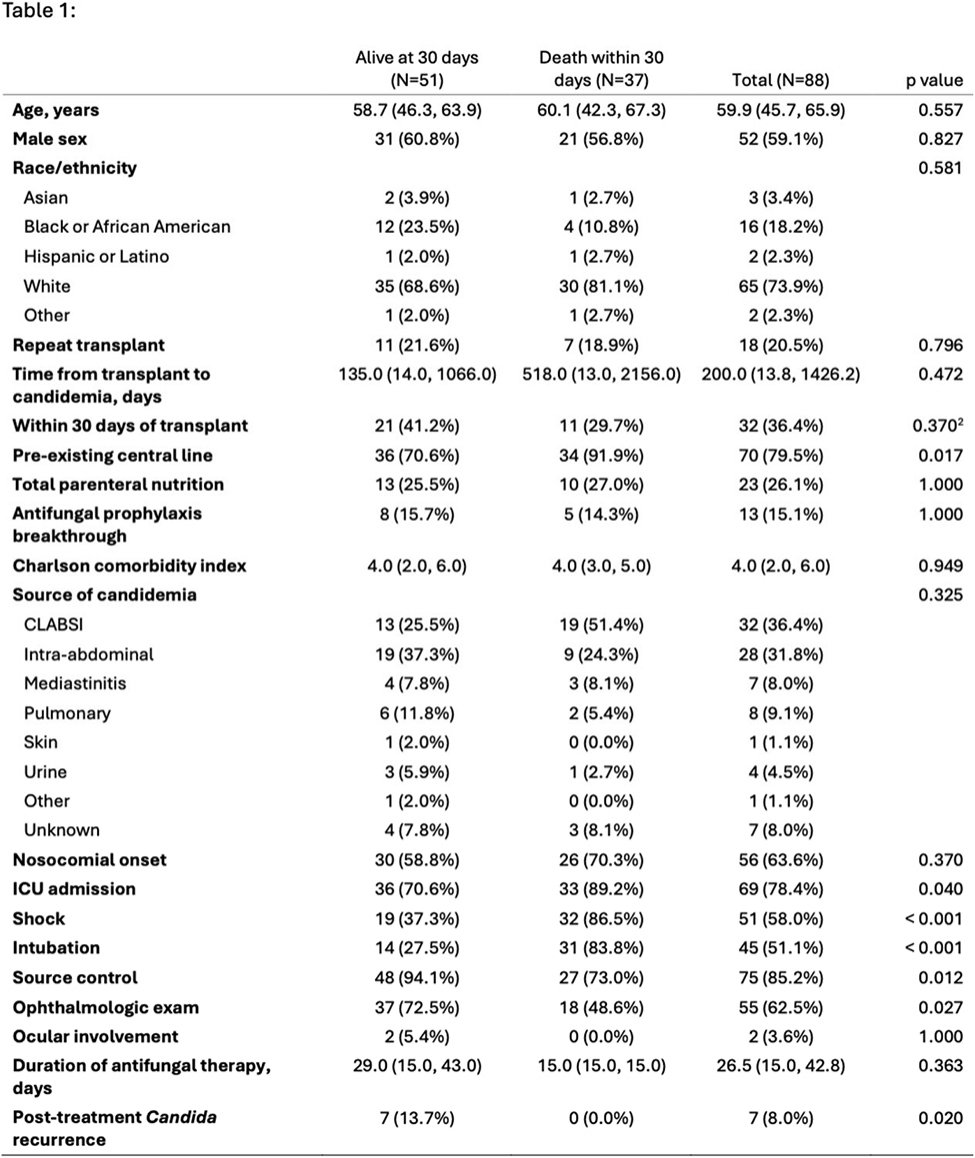
Baseline characteristics of participants.

**Figure 1:**
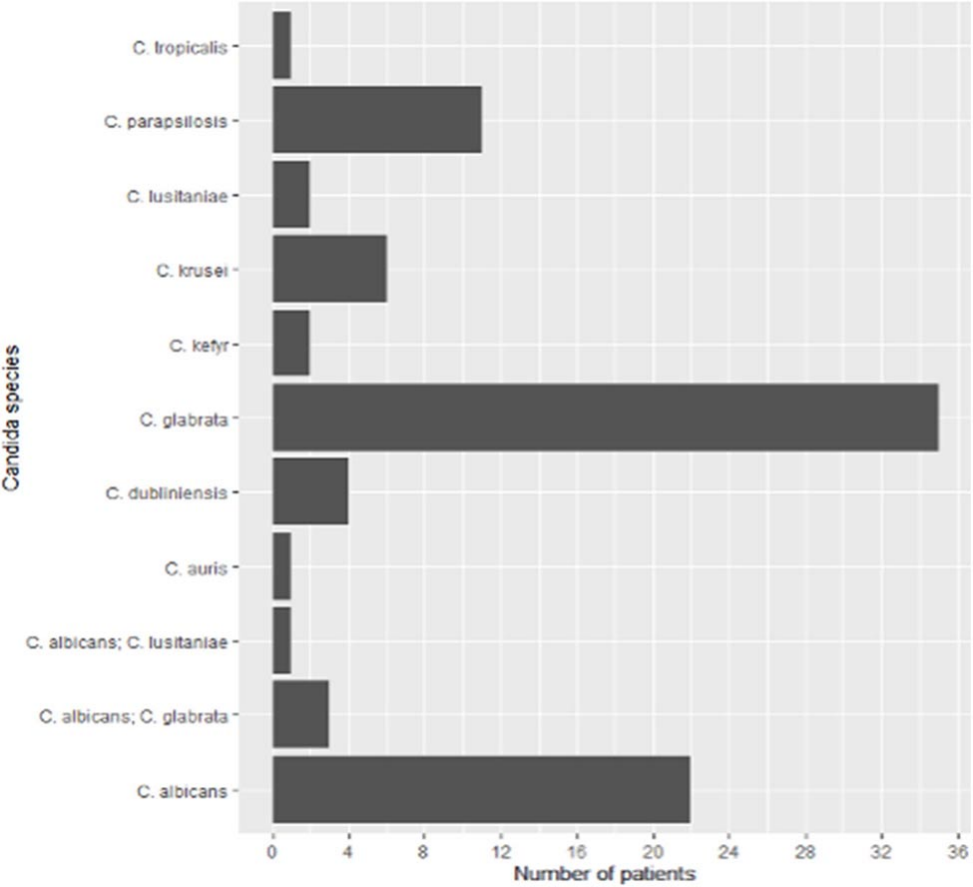
Distribution of Candida Species. Figure 1 represents the distribution of candida species isolated in the study cases.

**Methods:**

A retrospective cohort study was conducted on adult SOT recipients diagnosed with candidemia between 1/1/2009 and 3/31/2024. Epidemiological characteristics were described and stratified by mortality within 30 days of diagnosis. Secondary outcomes included ocular candidiasis and post-transplant *Candida* recurrence. Survival analysis was performed to investigate the factors associated with mortality and depicted using Kaplan-Meier curves.

**Figure 2:**
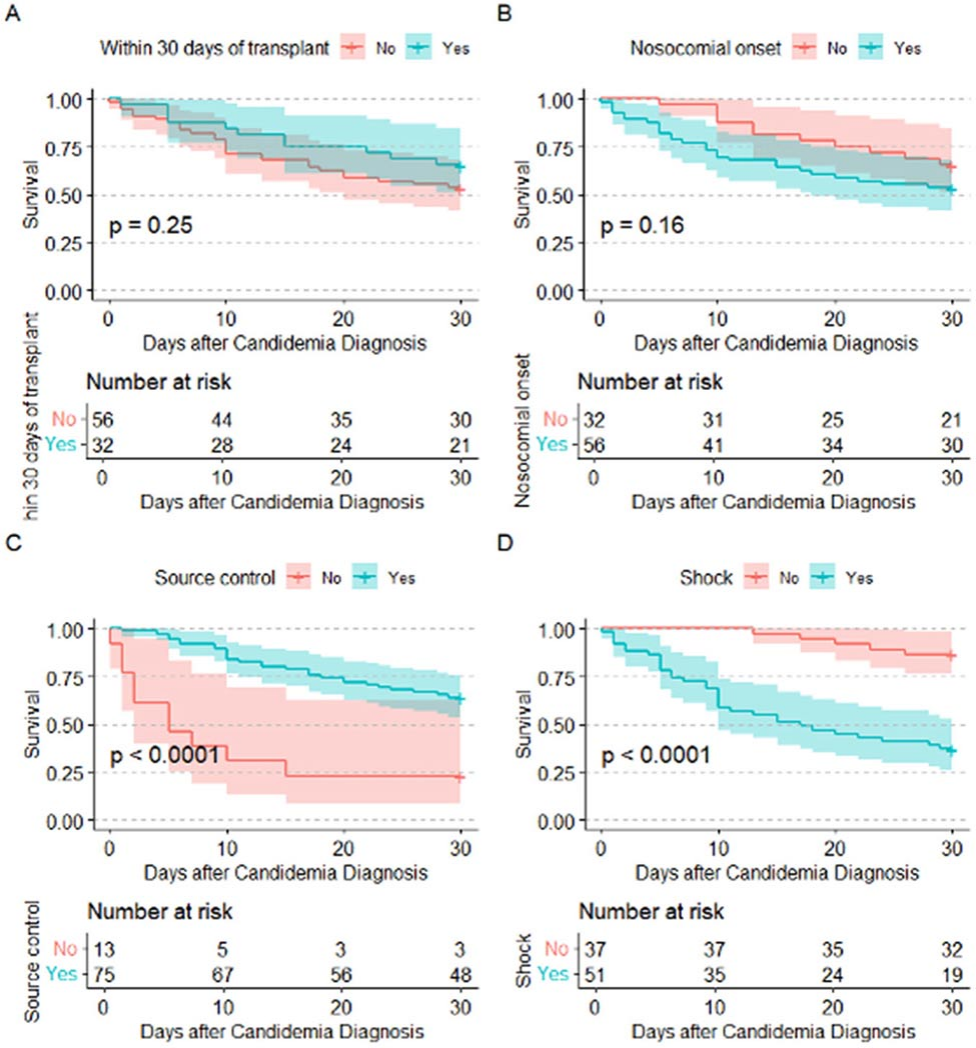
Factors Influencing Mortality in SOT Recipients with Candidemia. Figure 2 depicts Kaplan Meier curves for potential factors influencing mortality in SOT Recipients with Candidemia (a) Time from Transplant, (b) Nosocomial Onset, (c) Shock, (d) Source Control.

**Results:**

We included 88 SOT recipients, including 11 heart, 10 intestinal/multivisceral, 14 kidney, 16 liver, 20 lung, 1 vascular allograft, 16 multiple-organ recipients. Epidemiologic characteristics are summarized in table 1. Median time to candidemia was 200 days post-transplant, with 36.4% occurring within the first 30 days. *C. glabrata* was most isolated (40%), followed by *C. albicans* (25%), and *C. parapsilosis* (12.5%; figure 1). Coinfection with two distinct candida species was also observed in 4 cases. Primary sources of infection were central line-associated bloodstream infection (CLABSI, 36.4%) and intra-abdominal sources (31.8%). Initial antifungal therapy was predominantly with micafungin (89.2%). Source control was obtained in most cases (85.2%). Most patients developed septic shock (58.0%), required intubation (51.1%), and ICU admission (78.4%). Two (3.6%) of 55 patients who underwent formal eye examination had signs of ocular candidiasis. 37 (42.0%) died within 30 days of diagnosis. Shock and lack of source control were associated with increased mortality, while time after SOT and nosocomial onset of candidemia were not (figure 2).

**Conclusion:**

Candidemia is a devastating complication following SOT. Despite aggressive antifungal therapy and source control, mortality is high. Severity of initial presentation and source control seem more predictive of survival rather than baseline characteristics. These findings emphasize the need for improved prophylaxis and treatments to improve outcomes in this vulnerable population.

**Disclosures:**

All Authors: No reported disclosures

